# Selective mutism: follow-up study 1 year after end of treatment

**DOI:** 10.1007/s00787-014-0620-1

**Published:** 2014-09-30

**Authors:** Beate Oerbeck, Murray B. Stein, Are H. Pripp, Hanne Kristensen

**Affiliations:** 1 Department of Mental Health and Addiction, Oslo University Hospital, Po box 4959, Nydalen, 0424 Oslo, Norway; 2 University of California, La Jolla, San Diego, CA USA; 3 Department of Biostatistics, Epidemiology and Health Economics, Oslo University Hospital, Oslo, Norway; 4 Centre for Child and Adolescent Mental Health, Eastern and Southern Norway, Oslo, Norway

**Keywords:** Selective mutism, Follow-up, Behavioural intervention, Social phobia, Childhood anxiety

## Abstract

Cognitive behavioral therapy (CBT) is generally considered the recommended approach for selective mutism (SM). Prospective follow-up studies of treated SM and predictors of outcome are scarce. We have developed a CBT home and school-based intervention for children with SM previously found to increase speech in a pilot efficacy study and in a randomized controlled treatment study. In the present report we provide outcome data 1 year after having completed the 6-month course of CBT for 24 children with SM, aged 3–9 years (mean age 6.5 years, 16 girls). Primary outcome measures were the teacher rated School Speech Questionnaire (SSQ) and diagnostic status. At follow-up, no significant decline was found on the SSQ scores. Age and severity of SM had a significant effect upon outcome, as measured by the SSQ. Eight children still fulfilled diagnostic criteria for SM, four were in remission, and 12 children were without diagnosis. Younger children improved more, as 78 % of the children aged 3–5 years did not have SM, compared with 33 % of children aged 6–9 years. Treatment gain was upheld at follow-up. Greater improvement in the younger children highlights the importance of an early intervention.

## Introduction

Children with selective mutism (SM) are characterized by a consistent lack of speech in specific social situations in which there is an expectation for speaking (e.g. school) despite speaking in other situations (e.g. at home) [1]. Age of onset is typically before age 5 years [2, 3]. SM is relatively rare, with a prevalence of about 0.7–0.8 % in childhood, somewhat more frequent in girls [4] and bilinguals [5].

Selective mutism (SM) has over the years been found to co-occur with other anxiety diagnoses (particularly social phobia) and with neurodevelopmental disorders [6–9]. SM is also reported to run in families, and a family history study of 38 children with SM reported a clear excess of the personality trait of taciturnity in 1st-, 2nd-, and 3rd-degree relatives [10]. Support for a familial relationship between generalized social phobia and SM was found in a large study of parents to children with SM (70 parent dyads) [11]. As a result of new knowledge, SM has been classified as an anxiety disorder in the DSM-5, although upheld as a separate diagnosis from social phobia due to frequent comorbid language delays/disorders [12].

SM is considered to be hard to treat, and both medication and psychosocial treatments have been tried. With regards to medication, a double-blind, placebo-controlled study of children with SM from 1994 found that those treated with fluoxetine (*n* = 6) were rated as significantly more improved than the non-medicated (*n* = 9) at the end of the study period. However, most children in both groups were still very symptomatic [13]. Similar findings were reported in a retrospective 6–8 months naturalistic follow-up study that included 17 children diagnosed with SM (16 with comorbid social phobia). Those who received treatment with Selective serotonin reuptake inhibitors (*n* = 10) showed greater improvement than unmedicated children (*n* = 7), but the diagnoses persisted in 16 of the children [14].

The psychosocial treatment literature for SM has been dominated by case studies or case series including a wide array of treatment approaches. Furthermore, data are scarce both on the short-and long term outcome and predictors of outcome. The few existing long term outcome studies are usually retrospective, with few details provided about the given treatment. Using retrospective patient records, persisting communication problems were found in a substantial portion of 45 children with SM in a follow-up study (mean 12 years) [15]. Although SM improved, a high rate of psychiatric disorders was found in 33 adults with a childhood SM diagnosis [16]. A severity indicator of SM, taciturnity in the family and, by trend, immigrant status, had an impact on psychopathology and symptomatic outcome in young adulthood. In a retrospective study of 25 children, 2 to 10 years after referral, those given individual programs with a behavioural component were more likely to have improved compared with those given standard school-based remedial programs. A further poor prognostic indicator was past or present mental illness in the immediate family [17]. In spite of the reported psychiatric comorbidity [6–9] in children with SM, comorbidity as a predictor for remission of SM has, to our knowledge not been examined. Concerning comorbidity as a predictor of outcome in Cognitive behavioral therapy (CBT) for childhood anxiety disorders, results are not conclusive to date. While a study of 173 children found that pretreatment comorbidity was not associated with differences in treatment outcome for the principal anxiety disorder diagnosis [18], another study (*n* = 124) found that both total-and non-anxiety comorbidity added to the prediction of diagnostic recovery [19].

In 2006, a comprehensive review of the psychosocial treatment literature stated with some caution that CBT was recommended for SM [20]. A study using a group CBT approach for children with SM (*n* = 5), that also included a second group for their parents (psychoeducation and advice on how to handle SM) administered over an 8-week period, reported symptom improvement post treatment in all participants [21]. An alternating treatment design was applied in nine children with SM showing greater effectiveness for exposure based practice compared with contingency management [22]. Children, parents, and teachers rated outcome in terms of words spoken, and the reported effect sizes suggested improvement by children and parents, with somewhat less favourable teacher ratings. An established cognitive behavioural treatment for childhood anxiety disorders [23] was used in a case study of an 8 year old boy with SM. Symptom improvement was notable after 21 sessions, and the gain was also maintained at 1 and 6 months follow-up appointments [24].

In recent years, CBT interventions especially adapted for children with SM have been elaborated. The behavioural components have been emphasized, as the symptom of muteness and the young age of onset of SM make the cognitive restructuring less feasible. An important factor related to treatment is that children with SM tend to be most symptomatic in the school environment [25], thus requiring extensive treatment involvement of and coordination with teachers. Furthermore, as children with SM often fail to speak to the therapist, a special strategy to secure early child engagement as well as parental involvement, is vital. Consequently, Lindsey Bergman developed an integrated behavioral therapy for SM [26]. This treatment was conducted at the clinic with parental participation using graduated exposure tasks to the feared stimuli/situation (e.g. verbal communication). Therapists also remained in communication with teachers to ensure relevance of exposure tasks at school. A pilot Randomized controlled study of this treatment (RCT) including 21 children (4–8 years of age) found improvement in number of words spoken at school compared to baseline (blind raters), although, significant group differences did not emerge. However, a significant increase of speech was found after treatment, with no change in wait-list controls, as rated by the teacher on the School speech questionnaire (SSQ) [4]. Furthermore, 67 % of treatment recipients no longer fulfilled criteria for SM, and clinical gains were maintained at 3 month follow-up [27]. Diagnostic comorbidity was not assessed in this study, but significant reductions were reported in social anxiety symptoms per parent, but not per teacher, report.

In contrast to this clinic based treatment, our adaption of CBT for SM was the development of a school-based intervention, as children with SM tend to be most symptomatic in this environment [25]. To promote rapport with the child, increase parental engagement, and train on procedures later to be used at preschool/school, we started the treatment at home (three sessions), where these children feel most safe. To decrease the often co-occurring social anxiety, we used defocused communication as a general treatment principle. Central components of defocused communication are: to sit beside rather than opposite the child; to create joint attention using an activity the child enjoys rather than focusing on the child; to ‘think aloud’ rather than asking the child direct questions; to give the child enough time to respond rather than talking for the child; to continue the dialogue even though the child does not respond verbally; and try to receive a verbal answer in a neutral way rather than praising the child. In line with recommendations [20], we chose psychoeducation and behavioural interventions. The psychoeducation (including information about SM, and how to use defocused communication) was given by phone with the teachers and parents together to obtain a mutual understanding of SM and the child’s level of functioning. The behavioural interventions consisted of stimulus fading in the form of gradually increased exposure, as well as contingency management (use of positive reinforcement for speaking behavior) to be applied in a joyful play activity inspired by the Selective Mutism Resource Manual [28]. The behavioural interventions took place at preschool/school twice a week (each lasting half an hour) and followed six defined modules/speaking levels according to the progress of the child. The parents participated in the first module, the teachers from modules III to VI and peers/classmates from modules IV to VI. Table 1 describes the six modules (a more thorough description of the intervention is available in the RCT study [29]).

**Table 1:** Predetermined treatment modules reflecting increasingly difficult speaking levels (I–VI) to be obtained in the preschool/school setting from the baseline level of zero at T1; does not speak to adults (as defined by the diagnosis of SM in this study)

Modules	Description of the goal to be obtained in each speaking level at school
I	Speaks to the therapist (T) in a separate room with parent (P) present
II	Speaks to T in a separate room without P present
III	Speaks to one teacher in a separate room with T present
IV	Speaks to other teachers (and children) in a separate room with T present
V	Speaks to teachers (and children) in some settings without T present (speaks to some, but not all adults and/or in some groups in the classroom, but not in all larger settings, such as full class
VI	Speaks to teachers and children in all settings without T present (normal speech, indistinguishable from other children)

We found a highly favourable treatment outcome in a pilot efficacy study of seven preschool children diagnosed with longstanding SM (mean 20 months) [30]. Six children spoke freely in all preschool settings after a mean of 14 weeks treatment, and treatment gains were maintained at follow-up 1 year after end of treatment. Four bilingual children were included in this study, counteracting bilingualism as a negative outcome predictor.

We also found a significant treatment effect in an RCT study, with no change in wait-list controls [29]. In this study 24 children, 3–9 years of age, with a principal diagnosis of SM (24 with comorbid social phobia, 16 with additional diagnoses), were randomized to 3 months of treatment (*n* = 12), or wait-list controls (*n* = 12). A time by age interaction favoured younger subjects.

After 3 months of waiting, the children in the wait-list group (*n* = 12) received the same treatment, thus rendering a total of 24 children who were treated for a maximum of 6 months by local therapists (*n* = 21) at community health clinics all over Southern Norway. The present article will focus on the follow-up results from this effectiveness study conducted 1 year after the end of 6 months treatment for these 24 children with the teacher-rated SSQ and diagnostic status as primary outcome measures.

Based on the follow-up results from our pilot study we did not expect a significant decline of effect at follow-up.

We also expected that the younger children would remain more improved, and that severity of SM would be associated with less symptom improvement (based on our previous studies, and the early retrospective follow-up literature, respectively).

Due to both the situational nature of SM, and our school-based intervention, we further hypothesized that the children would show increased speaking behaviour primarily on the SMQ school subscale, and not on the public or at home subscales.

We also wanted to investigate whether diagnostic comorbidity and familial SM had an impact. As our treatment addresses SM, with the use of defocused communication to decrease social anxiety, we did not expect a treatment effect on the high rate of comorbid psychiatric disorders, other than possibly social phobia. We did, however expect a negative effect of familial SM, based on the early retrospective follow-up literature.

## Method

### Design

This is a follow-up study conducted 1 year after the end of a cognitive behavioural treatment adapted for children with SM with data from the follow-up (T4), as well as from baseline (T1), and after three- (T2) and six (T3) months of treatment. No additional therapy was given before follow-up. Table 2 presents an overview of informants and measures at T1 through to T4.

**Table 2:** Overview of informants and measures used at baseline (T1), after three- (T2) and six (T3) months of treatment, and 1 year after end of treatment (T4)

Informants	Time points for data collection			
	T1	T2	T3	T4
Teacher	SSQ	SSQ	SSQ	SSQ
Mother	ADIS-IV; SM module, K-SADS-PL			ADIS-IV; SM module, K-SADS-PL
Mother	CGI-Severity		CGI-improvement	CGI-improvement
Mother	SMQ	SMQ	SMQ	SMQ
Mother				Life events
Mother				User satisfaction

*SSQ* school speech questionnaire, *ADIS* anxiety disorders interview schedule (ADIS-IV), *K*-*SADS*-*PL* schedule for affective disorders and schizophrenia for school-aged children: present and lifetime version, *CGI* clinical global impression scale, *SMQ* selective mutism questionnaire, *Life Events* life events questionnaire, *User Satisfaction* from a National Examination of Parental Satisfaction with Treatment at CAHMS

### Participants

The sample consists of 24 children with SM, 3–9 years of age [16 girls, mean age 6.5 years (sd 2.0), nine children were in preschool; age 3–5 years, 15 were school children; age 6–9 years]. Six children were bilingual. At inclusion, nonverbal IQ and receptive vocabulary (mean = 100, sd = 15) was within the average range (mean/sd: 98/10 and 95/9, respectively, see our RCT study [29] for further details). We asked specifically about SM in the following family members: parents, siblings, grandparents, aunts/uncles, cousins, as well as in more distant family members. 10 of the 24 families had a positive history of SM in family members (in parents: *n* = 4, parents and siblings *n* = 1, grandparents, aunts/uncles *n* = 5). In 23 families, one or both parents described the presence of social anxiety symptoms in their own childhood.

#### Inclusion criteria

Children aged 3–9 years, consecutively referred for SM from outpatient Child and Adolescent Mental Health Clinics (CAMHS) or school psychology services in Southern Norway who fulfilled DSM-IV diagnostic criteria for SM. In addition, we specified that the children should not speak to adults in preschool/school, and that mutism was present also in the native language for bilingual children.

#### Exclusion criteria

(1) Parents who did not speak Norwegian or (2) children with IQ <50, psychosis or a pervasive developmental disorder. (3) Children on psychotropic medication or receiving another active treatment for SM. This resulted in 34 age appropriate referrals. A screening procedure, including anamnestic information and the Selective Mutism Questionnaire (SMQ) [25] excluded ten children [due to pervasive developmental disorder (*n* = 2), no mutism in native language (*n* = 1), use of speech to some adults in preschool/school (*n* = 7)]. The final inclusion of the 24 children was based upon a confirmation of the SM diagnosis after a home visit with a parental diagnostic interview and a child assessment to rule out severe intellectual problems.

### Therapist recruitment and training

The 24 children in the present study were at inclusion registered at a local CAMHS who then selected a local therapist resulting in a total of 21 clinically experienced therapists. All therapists had an advanced degree in mental health (master level), including clinical/educational therapists (*n* = 14), psychologists (*n* = 4), child psychiatrist (*n* = 1), nurses (*n* = 2). All but one therapist had a minimum of 5 years of clinical experience including no (*n* = 4), some (*n* = 11), or extensive (*n* = 6) previous work with selectively mute children. None had specific CBT training. They used our detailed manual describing defocused communication as a general treatment principle and the behavioural interventions under close guidance and supervision related to each session by phone from the first or last author, with no further treatment adherence measures.

Assessment instruments (see Table 2 for an overview of measures used at T1–T4).

#### Diagnosis of SM and comorbid diagnoses

SM was diagnosed using the SM module from the semi-structured anxiety disorders interview schedule (ADIS-IV) [31] with good construct validity [32]. The SM module relates to the speaking behaviour of the child in different social situations. In addition, we gathered detailed information on whether the child talked to adults in the preschool/school. To assess diagnostic comorbidity, we used the revised version of the schedule for affective disorders and schizophrenia for school-aged children: present and lifetime version (K-SADS-PL) [33]. Nine children below age 6 years were included, but adequate diagnoses can be made as long as the behavioural concepts and the understanding of life interference is adapted to be relevant to a preschool child [34]. Interviews were conducted by the last author, with extensive experience with ADIS/K-SADS interviews from research and clinical work, thus the diagnostic assessment at follow-up was not blinded.

#### A measure of global impairment and improvement

We used a mother-rated clinical global impression scale (CGI) [35], with a baseline rating indicating the severity of illness (CGI-S) and a later improvement rating (CGI-I). The CGI-S is a seven-point scale from: 1 = not at all a problem; 2 = minimal problem; 3 = mild problem with some impact on the child’s functioning; 4 = moderate problem with an impact on the child’s functioning; 5 = marked problem that limits the functioning of the child; 6 = severe problem, the child can only function with help; and 7 = very severe problem, not functioning. The CGI-I also uses a seven-point scale that describes the improvement/worsening of symptoms relative to baseline: 1 = very much improved; 2 = much improved; 3 = minimally improved; 4 = no change; 5 = minimally worse; 6 = much worse; and 7 = very much worse.

#### SM questionnaires

##### The School Speech Questionnaire (SSQ) [4]

Our primary outcome measure was the SSQ (based on speech frequency in the school context) rated by the child’s teacher at T1 to T4, as it was expected that teachers would have the most accurate information on speaking behaviour in this setting. The SSQ, a quantitative measure with no cutoff score, includes ten questions and is modified from the SMQ (see below) with acceptable internal consistency. Six of the SSQ questions (identical to the SMQ) are used to compute a mean score (range = 0–3), computed as the mean of the valid items, if at least half the items were valid. As in the SMQ, 0 indicates that speaking behaviour never occurs, and 1, 2 and 3 refer to seldom, often and always speaking, respectively. We used the Norwegian translation with permission from Lindsey Bergman, the developer of the measure. Internal consistency was somewhat low, but acceptable (*α* = 0.64).

##### The Selective Mutism Questionnaire (SMQ) [25]

The (SMQ) was rated by mothers at the same time-points (T1–4) for two reasons; to have multiple raters of speaking behaviour in the preschool/school setting, and to look at possible changes in two additional settings (at home and in public). The SMQ includes 32 questions scored from 0–3, where 0 indicates that speaking behaviour never occurs, and 1, 2 and 3 refer to seldom, often and always speaking, respectively. 17 of the SMQ questions are used to compute three subscale mean scores [preschool/school (six items), at home (six items) and in public (five items)] with the same 0–3 scoring range, computed as the mean of the valid items, if at least half the items were valid. The SMQ total factor score was computed from the sum of three subscales divided by three. In this study, one child had one missing item on one subscale. We used the Norwegian translation with permission from Lindsey Bergman, the developer of the measure. Acceptable internal consistency was found for the three subscales and the total score, respectively (*α* = 0.68, 0.73, 0.76, 0.77).

The SMQ has no cut-off score, but a psychometric study suggests a score ≤0.5 on the School and Public SMQ subscales for children with SM compared to ≥2.5 for children without SM [25].

##### Additional measures at follow-up (T4)

The mothers completed a modified version of the Life events questionnaire for adolescents [36] comprising 37 statements about the child’s life events from T3 to T4. Among the 37 statements, 25 are considered negative life events, 12 positive/ambiguous. One is asked to indicate whether each event had occurred or not, and to indicate an overall impact of the reported events combined on a scale from 0 (no distress) to 3 (severely distressed).

The mothers also completed a measure of parental satisfaction with the treatment previously used in a national CAMHS examination [37]. Presented here are the two questions pertaining to the overall satisfaction/dissatisfaction with the treatment offered to the child, and to how the parents were treated as caretakers during the process. The questions were rated on a scale from 1 (very dissatisfied) to 5 (very satisfied).

### Ethical approval

Written informed consent was provided by the parents. The study was granted approval by the Norwegian Social Science Data Services and the Regional Committees for Medical and Health Research Ethics.

### Missing data

There were no missing data on T1–T4. One child only received treatment for 3 months due to travelling abroad, but completed all assessments.

### Data analysis

Descriptive statistics using mean (standard deviation) or number of patients are presented for the SM questionnaires (SSQ, SMQ), the CGI, life events, user satisfaction and the diagnoses.

A linear mixed model for repeated measurements was applied to investigate the questionnaire total- and subscale scores from baseline (T1), 3 months (T2), 6 months (T3), and follow up 1 year after end of treatment (T4). Effect of age at diagnosis and severity of SM was examined as covariates. Mean differences between the four time points (T1–T4) were tested using Bonferroni corrections. The level of significance was defined as *p* < 0.05. As we found a low number of life events from T3 to T4 (mean 2.0, sd 1.86) not indicated by mother to represent any distress (mean 0.70, sd 0.80), life events were not included in the model.

A one-way ANOVA with Tukey post hoc comparisons was used to test for differences in the CGI-Improvement rating at follow-up (T4) between the SM related diagnostic groups (Diagnosed with SM, SM in remission, No SM, respectively).

## Results

### Questionnaire data

On our primary outcome measure, the teacher-rated SSQ, there was no significant decline of effect at follow-up. On the contrary, we found a small but significant increase in scores over time (*F*_3,69_ = 16,055, *p* < 0.001), indicative of further improvement. The significant increase took place from T1 to T2 [mean difference 0.52 (95 % CI 0.17–0.87, *p* = 0.001)]. Mean scores on the SSQ are presented in Table 3.

**Table 3:** SM questionnaires with data from baseline (T1) through to follow-up 1 year after end of treatment (T4)

Informant	Measure	T1 baseline mean (sd)	T2 At 3-months mean (sd)	T3 end, at 6-months mean (sd)	T4 follow-up mean (sd)
Teacher	SSQ	0.55 (0.43)	1.07 (0.83)	1.25 (0.86)	1.38 (0.78)
Mother	SMQ-school	0.50 (0.40)	1.03 (0.70)	1.23 (0.80)	1.47 (0.74)
	SMQ-at home	1.65 (0.64)	2.11 (0.47)	2.19 (0.57)	2.42 (0.45)
	SMQ-in public	0.33 (0.43)	0.63 (0.53)	0.88 (0.70)	1.04 (0.74)
	SMQ-total score	0.86 (0.35)	1.30 (0.45)	1.47 (0.57)	1.68 (0.55)
	CGI-severity	4.17 (1.05)			
	CGI-improvement			2.75 (1.03)	2.04 (0.91)

*SSQ* school speech questionnaire, *SMQ* selective mutism questionnaire, *CGI* clinical global impression scale including a severity- and an improvement rating

School Speech Questionnaire (SSQ) results further indicated a more pronounced increase in speech in younger children. In the model that also included age as a covariate and a time by age interaction, there was a significant effect of age (*F*_1,22_ = 4.843, *p* = 0.039) and a time by age interaction (a steeper increase of SSQ with time in younger children) (*F*_3,66_ = 5.016, *p* = 0.003), but still significant for time (*F*_3,66_ = 10.708, *p* < 0.001). Finally, we found a significant effect of SM severity (less effect in more severe cases) (*F*_1,21_ = 12.492, *p* = 0.002), as measured by SSQ scores at diagnosis, when it was included in the model. However, a significant effect of time (*F*_1,21_ = 17.597, *p* < 0.001), age (*F*_1,21_ = 7.925, *p* = 0.010) and time by age interaction (*F*_3,66_ = 5.018, *p* = 0.003) was still present.

The SSQ findings were in general replicated on the mother-rated SMQ. The SMQ total score showed a significant increase in scores over time (*F*_3,69_ = 28.494, *p* < 0.001) with the most pronounced increase from T1–T2 (mean difference 0.44 [95 % CI 0.19–0.69, *p* < 0.001]). However, increased speech was not restricted to the school setting. Using the SMQ subscale scores, measuring speaking behaviour at school, at home and in public, respectively, they all showed significant increases over time (*p* < 0.001, statistics not shown). See Table 3 for mean SMQ scores. The effect of age on the SMQ total score was not significant and therefore not included in this model. A borderline significance (*p* = 0.0053) of age was found on the SMQ school subscale, with no age effect on the other two subscales measuring speech behaviour at home and in public (*p* > 0.62).

### Diagnostic status and clinical global improvement

At follow-up, 12 children (50 %) no longer fulfilled diagnostic criteria for selective mutism (SM), as they spoke freely at school (nine of the 16 girls and three of the eight boys). Another four children spoke freely in some, but not all settings at school, and/or to some, but not all adults. Thus, rigorously speaking they did not fulfil the DSM-IV criteria of “Consistent lack of speech” and were categorized as SM in remission. The remaining eight children fulfilled diagnostic criteria for SM. A one-way ANOVA showed that these three diagnostic groups differed significantly on the CGI-Improvement scale (*F*_2,21_ = 15.19, *p* < 0.001). Tukey post hoc comparisons indicated that the eight children diagnosed with SM at follow-up (*M* = 3.0 95 % CI 2.37–3.63) differed significantly, both from the four children with SM in remission (*M* = 1.75, 95 % CI 0.95–2.55, *p* = 0.008), and from the 12 children with no SM (*M* = 1.50, 95 % CI 1.17–1.83 *p* < 0*.001*). The difference between the children with SM in remission and children with no SM was not significant (mean 1.75 and 1.50, respectively, *p* = 0.58).

Using the diagnoses, we again found a more prominent improvement in the younger children, as seven of nine children (78 %) aged 3–5 years at inclusion did not fulfil criteria for SM at follow up, compared with five of fifteen children (33 %), 6–9 years of age at inclusion. Pretreatment comorbidity had no impact on remission of SM at follow-up, as 50 % of both baseline noncomorbid (*N* = 8) and comorbid (*N* = 16) participants were diagnosed without SM.

At follow-up, comorbid anxiety diagnoses, as assessed by KSADS, were still frequent. Diagnoses other than social phobia were evenly distributed in children with and without SM at follow-up. In five children diagnosed without SM, there was also remission of social phobia (see Table 4). There was no negative effect of having SM in the family, as five of the ten children with familial SM (50 %) did not fulfill criteria for SM at follow-up.

**Table 4:** Diagnostic status at baseline (T1) and at follow-up (T4)

DSM-IV diagnoses	T1 (*N* = 24)	T4 (*N* = 24)	With SM at T4 (*N* = 12)	No SM at T4 (*N* = 12)
Selective mutism	*N* = 24	*N* = 12	12	0
Social phobia	*N* = 24	*N* = 19	12	7
Separation anxiety	*N* = 7	*N* = 4	3	1
Specific phobia	*N* = 6	*N* = 2	1	1
Generalized anxiety disorder	*N* = 2	*N* = 3	2	1
OCD	*N* = 2	*N* = 0	0	0
Tics	*N* = 2	*N* = 1	0	1
Enuresis	*N* = 6	*N* = 3	2	1
Encopresis	*N* = 1	*N* = 0	0	0
Total N of children with diagnoses other than SM and social phobia	*N* = 16	*N* = 11	5	6

Column 3 and 4 present diagnoses in children with (*N* = 12), and without (*N* = 12) SM at T4, respectively

### User satisfaction

The mothers reported an overall satisfaction with the treatment offered to the child [mean 4, 7 (sd 0.5), range 3–5], and with how the parents were treated as caretakers in the process [mean 4.9, (sd 0.3), range 4–5].

## Discussion

To our knowledge, this is the first prospective follow-up study conducted 1 year after the end of a cognitive behavioural treatment for children with SM in a reasonably large sample (in the context of prior SM studies).

As hypothesized, treatment gains were maintained at follow-up (T4). Furthermore, we found, as expected that younger children had greater improvement. This was shown both on our primary outcome measure (the teacher- rated SSQ), and by the fact that a greater proportion of the younger children (aged 3–5 years) no longer met diagnostic criteria for SM. As expected, we also found that severity of SM, as measured by SSQ at baseline, had a significant effect upon outcome at follow-up, thus confirming findings from a retrospective long term outcome study [16]. This finding is also in line with recent findings from the large childhood anxiety disorder treatment study (CAMS) [38]. However, although the participating children had a high load of familial social phobia and SM, in line with retrospective follow-up studies [10, 15], we could not confirm the effect of familial SM upon outcome in the present study.

Contrary to our expectations, significantly improved speaking ratings were reported on all SMQ subscales, not only on the school subscale, as found in our previous studies [29, 30]. Due to a possibly less entrenched mutism in younger subjects, it seems plausible that a younger age at inclusion seems to predict more improvement in the preschool/school environment. Furthermore, an effect of age at inclusion is congruent with the earliest SM literature suggesting that an early intervention may have been particularly important for those who improved with treatment [6, 39]. As the effect of age at inclusion was not examined in the Bergman study [27], we cannot compare our findings on this matter.

However, compared with the Bergman study [27], we had a smaller percent who no longer fulfilled criteria for SM (50 versus 67 %, respectively). This could simply reflect weaker results, as the children in our study were treated by local therapists, without prior CBT experience. In the Bergman study, the therapists were CBT trained, working at one clinic under direct guidance by the principle investigator, resulting in an excellent treatment adherence. Unfortunately, no formal treatment adherence measure was included in the present study, excluding the assessment of possible outcome variance due to therapist differences in adherence.

Our results could also be influenced by other factors. First, our sample was approximately 1 year older, and seemed to have a more severe SM (as measured by lower SSQ at inclusion (mean 0.55 versus 0.81, respectively).

To define what constitutes a clinically meaningful symptom improvement, and the definition of the criteria for SM diagnosis, are both challenging issues. First, the present study found a significant statistical improvement in speaking behaviour. One could expect that the level of speaking should approach a normal speaking behavior to represent clinical meaningfulness. We found that the mean teacher reported SSQ results changed from a level between never to seldom (0.55) to a level between seldom to often (1.38). Although lower than what is expected in children without SM (a level between often to always), we would argue that this change represents a clinically significant improvement for a child with SM. Secondly, concerning the diagnostic criteria of SM, the DSM does not specify whether the “Consistent lack of speech” means that some speaking in class (like in smaller groups of students, alone with teacher, or with teachers and children in smaller groups) is sufficient to avoid SM diagnosis. In the present study, we chose to categorize the four children who at follow-up spoke freely in some, but not all school settings as still having SM, but in remission. Due to their need for accommodation from others in some situations and the resulting lack of independence, we considered this represented an impairment qualifying for a diagnosis. However, an important point is that the CGI-improvement score at follow-up indicated that these four children were more similar to the children who no longer fulfilled diagnostic criteria for SM (*N* = 12), than to the children who were diagnosed with a definite SM (*N* = 8). If these four children were assigned to the no SM group, our results were comparable to the Bergman study. These issues also speak to the need for further refinement of the diagnostic criteria for SM. Even in the DSM-5 [12], it is not clear whether children with consistent lack of speech in some areas, but not others, would be considered to meet diagnostic criteria or not.

In line with a study on CBT for child anxiety disorders [18], pretreatment comorbidity was not associated with differences in remission of SM after treatment. As expected, our intervention did not have an effect upon comorbid psychiatric diagnoses, which were frequent at both T1 and T4. Interestingly, however, five children who no longer fulfilled criteria for SM also showed remission of social phobia (see Table 4), consistent with the notion that for many children with comorbid SM and social phobia, these two “disorders” are one in the same.

Although the present study found a small, but steady improvement over time, the most improvement took place from T1 to T2 (after 3 months of treatment). Interestingly, this finding seems to be in line with the reported mean results in the Bergman study. We cannot say for sure whether this is to be expected, whether therapy could end at this time point, or whether it indicates that something else, or a more intensified treatment is necessary from T2. One could also always question whether the increased speech observed after 3 months is a result of treatment or not. A strong indication for the effect of treatment is the lack of change after 3 months in the waitlist controls in the two RCT-studies to date [27, 29]. The children in the present study were not reported to have experienced much in the way of stressful life events from T3 to T4, or to have received any other form of treatment in the year following end of therapy with a likely effect on functioning.

Although the present study can show a significantly increased speech in the group as a whole, half the subjects continued to fulfill diagnostic criteria for SM. Comorbid anxiety disorders were still frequent, both in participants with and without SM. This study only included an intervention targeted specifically at SM. Whether a more broad intervention, including in particular specific exposure tasks for social phobia would be more effective, remains to be seen. Nonetheless, this study demonstrates that a cognitive behavioural school-based treatment can be effective for SM in children aged 3–9 years. The therapists used treatment including defocused communication as a general treatment principle, and a home and school-based intervention with gradual exposure to the feared situations in which speech is expected. Particularly promising is the fact that we could observe a significant effect in the hands of therapists who were not experts in SM. In addition, the favourable user satisfaction points to the feasibility of the intervention. Future research is needed to ascertain the active treatment components. For older children with SM, how to best utilize available school resources seems crucial to explore further, as also pointed out in the Bergman study [27]. Given the favourable results from this clinic-based study, we could hardly claim that a school-based intervention, such as ours, is necessary when treating SM. However, it gives direct access to the most important people (children and adults at school) related to the training of the child’s speaking behavior, and one form of a particularly close cooperation with the school, is underscored in both studies. Another similarity is the emphasis put on child and parent engagement, and the use of gradually exposure to the feared stimulus (e.g. speaking).

## Limitations

The sample size is a limitation. Second, the outcome raters were not blind to whether treatment had taken place. Third, the lack of a blind assessment of treatment adherence is also a limitation.

Finally, in the present study, we chose a school based intervention, that is to work at the arena where the symptoms are most pronounced and thus most impairing for the child. However, this might represent a limitation, as this treatment may not be easily translated to a general practice in the office settings.

## Conclusions

This is the first prospective follow-up study conducted 1 year after end of a cognitive behavioural treatment for children with SM, in a reasonably large sample (e.g. in the context of SM). Clinical gains were maintained at follow-up. Although increased speech and global improvement was found after treatment, a substantial number of children continued to meet criteria for SM, and comorbid diagnoses were still frequent.
